# Efficacy of Smartphone-Based Digital Brief Behavioral Treatment for Insomnia with Adjunct Light Therapy in Young Adults with Insomnia Symptoms and Late Chronotypes: A Three-Arm Randomized Controlled Trial Protocol

**DOI:** 10.3390/healthcare14101386

**Published:** 2026-05-19

**Authors:** Ryuji Furihata, Tomonari Shimamoto, Yukako Nakagami, Satoe Okabayashi, Kosuke Kiyohara, Toshiki Akahoshi, Yoshimitsu Takahashi, Taku Iwami

**Affiliations:** 1 Agency for Student Support and Disability Resources, Kyoto University, Yoshida-honmachi, Sakyo-ku, Kyoto 606-8501, Japan; 2 Department of Preventive Services, Kyoto University School of Public Health, Graduate School of Medicine, Kyoto 606-8501, Japan; 3 Agency for Health, Safety and Environment, Kyoto University, Kyoto 606-8501, Japan; 4 Department of Food Science, Otsuma Women’s University, Tokyo 102-8357, Japan; 5 Shinjuku Sleep and Respiratory Clinic, KEISHINKINENKAI Medical Corporation, Tokyo 160-0023, Japan; 6 Division of Respiratory Medicine, Department of Internal Medicine, Nihon University, Tokyo 101-8309, Japan; 7 Department of Implementation Science in Public Health, Kyoto University School of Public Health, Kyoto 606-8501, Japan

**Keywords:** digital, brief behavioral treatment for insomnia, chronotype, insomnia, light therapy, randomized–controlled trial, smartphone app

## Abstract

**Background:** Insomnia symptoms associated with late chronotypes are prevalent among young adults, and frequently lead to sleep deprivation, morning-awakening difficulties, and excessive daytime sleepiness. These conditions are associated with psychological distress, impaired academic functioning, and reduced self-esteem. Although chronobiological behavioral interventions are effective, their accessibility remains limited. To bridge this gap, we developed a smartphone application delivering digital brief behavioral treatment for insomnia (BBT-I). We also designed a four-week program integrating the app with light therapy (LT) via wearable glasses named “Digital BBT-I + LT”. This randomized controlled trial will evaluate the efficacy of digital BBT-I with LT and digital BBT-I alone compared with a waitlist control group. **Methods:** This three-arm parallel-group randomized controlled trial will target young adults with insomnia symptoms and late chronotypes. Participants will be randomized to receive digital BBT-I with adjunctive LT, digital BBT-I alone, or a waitlist control condition. The primary outcome will be insomnia symptom severity, assessed using the Insomnia Severity Index. Evaluations will be conducted at baseline, weekly during the four-week intervention period (days 8, 15, 22, and 29), and at the 3-month follow-up. **Discussion:** Late chronotypes and insomnia significantly impair sleep and mental health in young adults. Despite the recognized importance of chronobiology-based treatments, they are rarely adopted as standard care. This highlights the need for scalable digital interventions, such as digital BBT-I. By rigorously assessing these strategies, this trial aims to provide clinical evidence regarding accessible interventions to improve sleep and mental health outcomes in this population.

## 1. Introduction

Sleep-related health challenges vary across the lifespan; however, shifts in sleep–wake rhythms have become a primary concern in young adults. In young adults, chronotypes shift sharply toward the evening, often leading to delayed sleep–wake phase disorder (DSWPD). Under these conditions, sleep patterns fall significantly behind socially required schedules, such as school start times, which are highly prevalent in this age group [1]. DSWPD causes difficulty falling asleep, difficulty waking up, sleep deprivation, and daytime sleepiness, which can further trigger psychological symptoms such as anxiety and depression. These difficulties often impair academic or occupational performance and erode self-esteem [2], representing a significant unmet medical need.

Insomnia accompanied by a late chronotype poses distinct clinical challenges. Individuals with delayed sleep timing frequently respond poorly to conventional hypnotic medications [3,4], and standard digital cognitive behavioral therapy for insomnia (CBT-I) has shown limited effectiveness in this population [5]. These shortcomings reflect a substantial unmet clinical need for interventions that directly target the circadian misalignment characteristic of late chronotypes. Clinical guidelines for DSWPD recommend chronobiological approaches—personalized sleep scheduling, psychoeducation, and post-awakening light therapy (LT) or timed melatonin [6]. However, face-to-face behavioral treatment is difficult to scale because of a shortage of trained specialists. To bridge this gap, scalable digital interventions that incorporate circadian-based strategies are needed. Combining brief behavioral treatment for insomnia (BBT-I) with LT offers a pragmatic, mechanism-driven approach that can address both behavioral and circadian contributors to insomnia in late chronotypes. Generation Z (“digital natives”) favors mobile-first, frictionless solutions for health management [7], making a smartphone-based platform particularly well-suited to deliver scalable, chronobiology-informed care.

On this basis, we developed the “Sleep Healthy” smartphone application to deliver digital BBT-I and paired it with wearable LT glasses to create a four-week “digital BBT-I + LT” program. A prior pilot randomized controlled trial (RCT) in university students demonstrated greater reductions in Insomnia Severity Index (ISI) scores and a shift toward morningness in the intervention group [8], but external validity remains to be established.

The present study, therefore, conducts a three-arm RCT comparing digital BBT I + LT, digital BBT I alone, and a waitlist control to evaluate efficacy in young adults with evening-type chronotypes. We primarily hypothesized that young adults with insomnia and late chronotypes in the digital BBT-I groups would achieve significantly greater reductions in ISI scores from baseline to post-test than the waitlist control group. Furthermore, we hypothesized that: (1) digital BBT-I with LT groups would show a significant significantly greater reductions in ISI scores from baseline to post-test than the waitlist control; (2) intervention groups would show a significant shift toward morningness (Morningness–Eveningness Questionnaire [MEQ] scores) by the post-test; (3) these groups would exhibit improved daytime sleepiness (Epworth Sleepiness Scale [ESS]), sleep health (Ru-SATED), depressive symptoms (Patient Health Questionnaire-9 [PHQ-9]), and sleep knowledge by the post-test; and (4) clinical gains would be sustained at the 3-month follow-up. The findings of this study are expected to contribute to the development of new prevention and treatment strategies for sleep-related problems among young adults.

## 2. Materials and Methods

### 2.1. Trial Design

This was a three-arm parallel RCT designed to evaluate and compare the effects of digital BBT-I using a smartphone application on sleep and mental health, relative to a waitlist control group.

Participants were randomized to one of three conditions: digital BBT-I with LT, digital BBT-I, or a waitlist control group. Participants completed an online questionnaire: a pre-test at baseline, a post-test after the 4-week intervention, and a 3-month follow-up survey. The ISI was measured weekly during the intervention period. An online questionnaire was used to measure insomnia symptoms, sleep health, mental health, and sleep knowledge.

### 2.2. Study Setting

The study was conducted in accordance with the Declaration of Helsinki and approved by the Ethics Committee of Kyoto University Graduate School and Faculty of Medicine (protocol code: C1776, date: 2 March 2026). This study was conducted at Kyoto University, Japan.

### 2.3. Recruitment and Participants

Participants from Kyoto University, Japan, were recruited through websites and flyers. Written informed consent was obtained from participants during online information sessions using a web conference system.

Individuals who met the following criteria were included in this study: (1) MEQ [9] score between 16 and 41, “obvious night owl (16–30)” or “almost night owl (31–41)”; (2) ISI ≥ 8 [10]; (3) age between 18 and 40 years at the time consent was obtained; (4) having a personal smartphone; (5) having Japanese as the first language; (6) owners of a smartphone whose version and model were compatible with the application.

We excluded individuals who met one or more of the following criteria: (1) time difference between the current average and target wake-up time exceeding 5 h; (2) those who may be subjected to significant time zone changes, such as international travel, during the study period; (3) shift and late-night workers; (4) those with photosensitivity; (5) those with a history of serious medical conditions; (6) those who underwent treatment for insomnia or sleep disorders; (7) individuals undergoing therapy at a psychiatric facility; or (8) those who responded that the frequency of “thoughts that you would be better off dead or of hurting yourself in some way” occurred more than half of the days in the previous two weeks for question 9 of the PHQ-9. These criteria were confirmed through online screening questionnaires and online information sessions.

After providing informed consent, participants completed an online interview to determine whether they met all the inclusion criteria and none of the exclusion criteria. Participants who met all the eligibility requirements completed the baseline questionnaires. They were randomized to one of three groups: digital BBT-I with LT, digital BBT-I, or a waitlist control condition. At the research office, participants assigned to either intervention group (digital BBT-I with LT or digital BBT-I) received instructions on downloading and using the Sleep Healthy app and were asked to complete a daily sleep diary using the app. Participants assigned to the digital BBT-I group, along with the LT group, received instructions on how to use light glasses. All study participants received a portable activity monitor at the research office and received instructions on how to use it. Ideally, the four-week intervention period began on Monday, following their visit to the research office. During the intervention period, participants completed a brief weekly questionnaire that included the ISI. All participants completed a post-test assessment at the end of the four-week intervention period.

The anticipated risk to the participants in this trial was considered minimal. Harms were assessed systematically using the following procedure:

Monthly Team Meetings: The principal investigator and site investigators met monthly to review the outcomes and adverse event data and monitor participant safety.

Weekly Monitoring: Participants’ symptoms and potential side effects were evaluated using weekly questionnaires throughout the intervention period.

Ethical Reporting: Investigators adhered to the ethical guidelines [11] for reporting adverse events, ensuring that any information affecting participant safety is communicated swiftly.

A Data Monitoring Committee (DMC) was not established for this trial. The reasons for this decision included the low risk associated with digital BBT-I and LT interventions, the relatively short study duration, and limited funding and personnel availability.

### 2.4. Study Assessment Instruments

Primary Outcome

Variable:

(1) **ISI**

The Japanese version of the ISI was administered [10]. This is a seven-item questionnaire with scores ranging from 0 (no insomnia) to 28 (severe insomnia). Individuals with scores ≥ 8 were defined as mild insomniacs, scores 15–21 as moderate insomniacs, scores 22–28 as severe insomniacs [10].

Secondary Outcomes

Variables:

(2) **MEQ**

The Japanese version of the MEQ was used [9]. The MEQ comprises 19 multifaceted questions on lifestyle characteristics and sleep habits. The overall score was used to determine whether a person was a morning or a night person. Individuals with scores of 70–86 were defined as definitely morning type, 59–69 as moderately morning type, 42–58 as intermediate or neither type, 31–41 as moderately evening type, and 16–30 as definitely evening type [9].

(3) **ESS**

The Japanese version of the ESS was also employed [12]. This is an eight-item questionnaire with scores ranging from 0 (no daytime sleepiness) to 24 (severe daytime sleepiness).

(4) **Ru-SATED**

The Japanese version of Ru-SATED was also used [13,14,15]. This six-item questionnaire evaluates multidimensional sleep health, with scores ranging from 0 (poor sleep health) to 12 (good sleep health).

(5) **PHQ-9**

The Japanese version of the PHQ-9 was used to assess depressive symptoms [16]. On a scale of 0 to 3, the respondents indicated the frequency with which they experienced the nine depressive symptoms. The total scores ranged from 0 to 27, with scores of ≥10 representing clinically significant depressive symptoms.

(6) **Sleep quiz**

A sleep quiz with ten “true or false” responses was used to assess the participants’ knowledge of sleep [8,17]. The questions covered topics such as electroencephalography, sleep stages, hormone secretion, caffeine intake, effects of bathing, effects of light, changes in body temperature during sleep, age-related sleep changes, and stimulus control methods. The total score (range 0–10) was calculated by summing the item scores.

(7) **Other measures**

The online questionnaire recorded sociodemographic information (age, sex), height, weight, cohabitation status, exercise frequency (≤3 or ≥3 times per week), alcohol consumption (never vs. sometimes or daily), smoking habits (yes or no), and intake of caffeinated beverages (coffee, tea, carbonated beverages) (yes or no). Body mass index (BMI) was calculated using the self-reported height and weight. Participants were divided into two groups: obese (BMI ≥ 25) and non-obese.

**Sleep Diary Measures**

Participants in the intervention group completed a daily sleep diary using the application. In the sleep diary, participants recorded the following: (1) the time of going to bed; (2) sleep onset latency; (3) number of awakenings; (4) duration of awakenings; (5) time of final awakening; (6) terminal wakefulness; (7) self-rated sleep quality; (8) duration of napping; (9) time of excessive daytime sleepiness; (10) whether the recording date was a weekday or a holiday; and (11) behavior before bedtime, including alcohol consumption, caffeine intake, smoking, and hypnotic medication use.

**Sleep Recording**

To identify participants who had extremely irregular sleep–wake rhythms and for whom sleep and wake times could not be determined, all participants recorded their activity during nighttime while the lights were off using the Sleep Sign Act (Estera Corporation, Saitama, Japan), which was worn on the waist [18]. Every 0.125 s, the number of times that acceleration exceeded a reference value was summed, and the value was recorded as the activity value over 2-min bins. The activity intensity is calculated from the activity value as a value from 0 to 31 (32 levels). An activity intensity of 0 means the subject did not move, and higher values indicate greater activity. The sleep data recorded by the Sleep Sign Act are stored on a password-protected local PC and are not imported into the “Sleep Healthy” app. This product does not perform validation during use.

### 2.5. Intervention

**Digital BBT-I**

Digital BBT-I was used as an intervention. Digital BBTI was administered using the Sleep Healthy application developed by Kyoto University. The application has the ability to set and notify an individualized sleep schedule for phase-advanced, the ability for participants to record sleep diaries and view summary sleep data, notification functions, and educational functions that provide sleep hygiene instructions, stimulus control therapy, and progressive muscle relaxation (PMR) in checklists, columns, videos, and quiz forms. The duration of the intervention program was 4 weeks.

(1) ***A sleep schedule for phase-advanced***

Prior to the start of the study, participants visited the research desk and entered their final target bedtime, wake-up time, current average bedtime, and wake-up time into the application with the help of the research staff. During this time, research staff advised participants to set a target wake-up and bedtime, recommending aiming for 8 h of sleep in their target schedule. The intervention group maintained their own daily rhythm while keeping a sleep diary during the first week. After the second week, the sleep phase was advanced, and the application individually notified them of their target bedtime for that day and the next morning. On weekdays, the sleep and wake times were advanced by 30 min based on the average bedtime and wake-up times [19], and on weekends, the same schedule as on Fridays was used. The application presented the target bedtime, wake-up time, and sleep duration on the notification screen daily.

(2) ***Sleep hygiene education, stimulus control therapy, and PMR***

For guidance on sleep hygiene and sleep knowledge [20], the application provided a checklist of sleep hygiene education points, sleep columns, videos, and sleep quizzes. We provided information on the behaviors recommended for stimulus control therapy and the theoretical background of the method in the form of sleep columns and sleep quizzes. A video explaining PMR was provided.

Between weeks 1 and 4, messages were sent on the notification screen recommending viewing columns and sleep quizzes for sleep education. During week one, notifications were sent to present a list of sleep hygiene instructions and to work on improving them. On day 13, notifications were sent to complete the exercises while watching a PMR video of approximately 7 min in length. All digital content in the application was made available to the participants in the intervention group at any time during the intervention period.

(3) ***Sleep Diary Measures***

The participants completed a daily sleep diary using the application [21]. Using the diary data, the application calculated the time in bed (time in bed = final rising time − time of going to bed), sleep time (total sleep time = time in bed − sleep onset latency − waking after sleep onset − bed out latency), sleep efficiency (sleep efficiency = [total sleep time/time in bed] × 100), and mid-sleep time (mid-sleep time = sleep onset time + (total sleep time)/2). In addition, the application presented these sleep variables numerically and graphically.

(4) *Data Security and Privacy*

To safeguard participant confidentiality and ensure compliance with the General Data Protection Regulation (GDPR), the Sleep Healthy application implements a multilayered security framework:

Encryption: All data transmissions are secured via HTTPS/TLS, and data stored in Firestore and Cloud Storage is protected through robust Google Cloud–managed encryption.

Access Control: User authentication is enforced through Firebase Authentication, while Firestore Security Rules restrict access to ensure that participants can view only their own records.

GDPR Compliance: In accordance with data minimization principles, only essential sleep-related variables are collected. Participants provide informed digital consent and may permanently delete their accounts and associated data at any time.

Anonymization: For research analyses, sleep data are anonymized by separating personally identifiable information from analytical datasets.

Monitoring and Transparency: System integrity is supported by continuous audit logging and a clear, accessible privacy policy.

This study included two intervention groups and one comparator group. The interventions were delivered over a four-week period.

*Intervention Group 1: Digital BBT-I + LT*

Digital BBT-I: delivered automatically via the “Sleep Healthy” smartphone application.

LT: After recording baseline sleep–wake rhythms during the first week, participants wore LED glasses (Pegasi 2.2) for 30 min immediately after waking up from weeks 2–4. These glasses emitted light at approximately 470 nm [22]. If glasses were unavailable, participants were instructed to receive natural sunlight by opening curtains immediately after waking up. The actual number of days of usage was recorded using a weekly questionnaire.

Previous research has shown that morning light advances the phase of the circadian rhythm [23], and it is generally recommended to be exposed to light for 30 min after waking up [24]. Moreover, light within the 420–500 nm wavelength range exerts a pronounced influence on the human circadian system. Short-wavelength light near 460 nm (blue light) produces the strongest suppression of endogenous melatonin at equivalent intensities. It induces the most substantial shifts in circadian phase, as well as notable effects on wakefulness and the pupillary light reflex. This wavelength-dependent sensitivity is referred to as the “action spectrum” [22].

*Intervention Group 2: Digital BBT-I*

Participants in this group received the same four-week digital BBT-I program via the “Sleep Healthy” app as Group 1.

Instead of using light glasses, participants were instructed to expose themselves to natural sunlight immediately upon waking.

*Comparator: Waitlist Control Group*

Participants in this group did not use smartphone applications during the study period and continued their usual daily activities. To ensure ethical standards and provide benefits, they were offered the opportunity to use the “Sleep Healthy” application after the study period had ended.

Administration and Monitoring

Initial Session: After obtaining informed consent, the research staff at the research office provided participants with instructions on how to download and use the “Sleep Healthy” app.

Activity Monitoring: All participants were provided with a portable activity monitor (Sleep Sign Act) worn on the waist to track nocturnal activity throughout the study.

Implementation: The digital BBT-I was delivered automatically by the application, and app engagement data were recorded. Participants’ adherence to LT was monitored through weekly questionnaires.

Additional Materials

The specific digital BBT-I protocol used in this study was identical to that implemented and validated in a previous pilot RCT targeting university students with insomnia and late chronotypes. Details of this protocol can be found in a published pilot study [8].

### 2.6. Randomization and Blinding

Participants were randomly assigned in a 1:1:1 ratio to the intervention (digital BBT-I with LT), intervention (digital BBT-I), or waitlist control group using stratified permuted-block randomization with randomly varying block sizes. Stratification was based on sex (male or female) and baseline ISI scores (<15 or ≥15 points [10]). The allocation sequence was generated using computer-generated random numbers. It was maintained solely by a third investigator (KK), who was not involved in participant recruitment, eligibility screening, baseline assessment, or intervention delivery. After eligibility confirmation, informed consent, and baseline assessment had been completed, the site investigators provided the third investigator with a list containing only participant IDs and stratification factors. The third investigator then assigned participants according to the pre-generated allocation sequence within each stratum and returned the group assignments to the research office. The allocation sequence, including the block sizes, was not disclosed to the site investigators. This centralized allocation procedure ensured that treatment allocation remained concealed from site investigators until participants had been enrolled and baseline assessment completed, thereby minimizing the risk of selection bias.

Owing to the nature of the interventions, participants and site investigators could not be blinded. However, to minimize bias, the data analysts (TS) remained blinded to group assignments until the primary analysis was completed.

### 2.7. Outcome Measures

The primary outcome of the protocol was the change in insomnia symptoms, as measured by the ISI. The ISI was measured at baseline (T_1_), once a week during the intervention (on days 8, 15, and 22), post-test (day 29) (T_post_), and again at the 3-month follow-up (T_3 month_).

The secondary outcomes were assessed using the MEQ, ESS, Ru-SATED, PHQ-9, sleep quiz, and other measures. They were measured at baseline, post-test (day 29), and at a 3-month follow-up.

**Figure 1:**
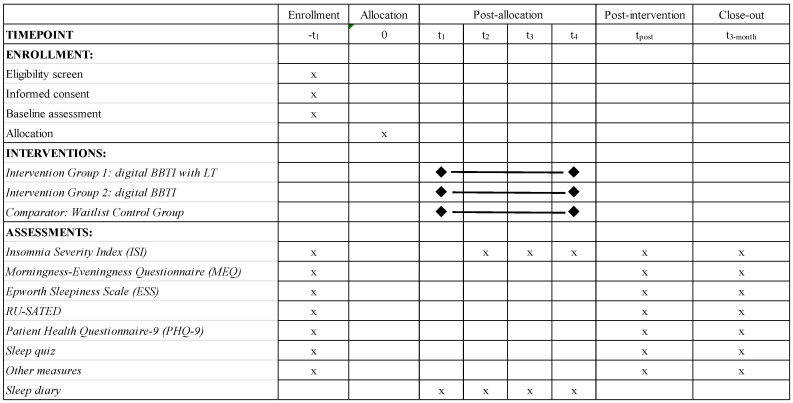
Schedule of enrollment, interventions, and assessments for study participants.

Figure 1 shows a synopsis of all the outcome measures and assessment time points.

## 3. Statistical Analysis

### 3.1. Sample Size

Sample size calculations were informed by the results of our pilot study [8] and the existing literature [25]. The pilot study, which utilized digital BBT-I + LT, demonstrated a large effect size on ISI scores (*d* = 1.080; 95% CI: 0.226–1.914). Additionally, a network meta-analysis reported a moderate effect size for digital CBT-I (*d* = −0.78; 95% CI: −1.18, −0.38) [25]. Based on these findings, we conservatively estimated a medium-to-large effect size of *d* = 0.80 for the comparison of digital BBT-I against the waitlist control.

To detect this effect size with 80% power and a two-sided α = 0.05, a total sample of 90 participants (30 per group: digital BBT-I + LT, digital BBT-I, and control) was required. This target sample size accounted for an estimated 20% attrition rate (i.e., 80% retention), ensuring that at least 25 participants per group would complete the post-test assessment.

### 3.2. Analysis

For the primary outcome (ISI), longitudinal data were analyzed using linear mixed-effects models with random intercepts to account for repeated measurements and individual variability. The fixed effects in the model included the treatment group, five time points (baseline and weeks 1–4), and the group-by-time interaction term. Secondary outcomes were compared using independent-samples *t*-tests to evaluate mean changes from baseline to post-test and 3-month follow-up. Harms and baseline covariates were summarized using descriptive statistics.

The analysis included all participants randomized according to their assigned groups. The use of linear mixed-effects models allowed the inclusion of all participants who provided at least some follow-up data, thus ensuring a robust assessment of the effectiveness of the intervention.

Missing data were handled using linear mixed-effects models, which automatically perform imputation based on available longitudinal data. This approach accounts for participants who may discontinue the intervention, while still allowing their available weekly measurements to contribute to the overall analysis.

While the primary focus is on the main hypotheses, subgroup analyses may be conducted if qualitative reviews suggest that specific patient subgroups, defined by demographic characteristics, differences in app engagement data, or variations in alliance [26], exhibit differing patterns of response to treatment.

### 3.3. Additional Statistical Considerations

Multiplicity: Multiplicity adjustment will not be applied to the single prespecified primary outcome. Secondary outcomes will be adjusted using the Holm procedure. Model assumptions and convergence were assessed using standard diagnostic procedures, including residual and Q–Q plots.

Missing data and sensitivity analyses: The primary analysis will use linear mixed-effects models under the missing-at-random assumption within an intention-to-treat framework. Sensitivity analyses will include per-protocol analyses and alternative covariance structures to assess robustness.

## 4. Discussion

Insomnia symptoms associated with late chronotypes are prevalent among young adults, and when left untreated or inadequately managed, they can lead to significant functional impairments. Therefore, it is critical to investigate the efficacy of interventions for insomnia related to delayed sleep–wake phases in this population. In addition to alleviating insomnia symptoms, such interventions have the potential to enhance overall sleep and mental well-being.

Although the efficacy of chronobiological behavioral treatments is well recognized [6], access to these interventions remains limited. Leveraging digital technology to expand the reach of these treatments could provide direct support to young adults and improve their overall well-being. This clinical trial aimed to evaluate two approaches to care for young adults experiencing insomnia with late chronotypes: digital BBT-I combined with LT using light glasses and digital BBT-I alone. Ultimately, this study aimed to generate new insights and clinical guidelines, providing evidence-based support for young adults struggling with sleep-related issues.

This study has several limitations. First, a questionnaire was used to assess insomnia symptoms and late chronotypes. Future clinical studies should be conducted in participants who meet the clinical diagnostic criteria for DSWPD, especially when the application is intended for use in medical settings. Second, because the study was conducted at a single university—even though it is a large institution—the characteristics of the participants may have been biased. To enhance generalizability, additional multicenter studies will be necessary. Third, due to the nature of the active behavioral components (digital BBT-I and LT), blinding participants and site investigators was not feasible, introducing the possibility of performance and measurement bias. We addressed these concerns through several methodological safeguards. To mitigate performance bias, the “Sleep Healthy” app provided a fully automated and standardized intervention flow, minimizing human contact and reducing the risk of undue investigator influence that can occur in face-to-face therapy. To address measurement bias, the primary outcome analysis was conducted by a statistician who was blinded to group allocation and had no involvement in participant recruitment or intervention delivery. While self-reported measures such as the ISI are susceptible to expectancy effects, their standardized format and established sensitivity to change help maintain consistency across groups. Moreover, there is a growing consensus among sleep researchers regarding the importance of subjective outcomes in sleep assessment.

## 5. Conclusions

The findings derived from this protocol aim to provide robust evidence for digital BBT-I in addressing insomnia symptoms associated with late chronotypes in young adults. This evidence will contribute to the broader integration of non-pharmacological interventions into standard healthcare systems. Furthermore, the rigorous methodology outlined in this study can serve as a benchmark for future research, fostering scientific reproducibility, adaptation to diverse healthcare contexts, and continuous refinement of digital health interventions.

## Data Availability

Researchers at Kyoto University will have access to the final trial dataset. De-identified participant data and the data dictionary will be made available upon reasonable request to the corresponding author, starting 6 months following publication.

## Abbreviations

The following abbreviations are used in this manuscript:

BBT-I	Brief Behavioral Treatment for Insomnia
LT	Light Therapy
DSWPD	Delayed Sleep–Wake Phase Disorder
CBT-I	Cognitive Behavioral Therapy for Insomnia
RCT	Randomized Controlled Trial
ISI	Insomnia Severity Index
MEQ	Morningness–Eveningness Questionnaire
ESS	Epworth Sleepiness Scale
PHQ-9	Patient Health Questionnaire-9
DMC	Data Monitoring Committee
PMR	Progressive Muscle Relaxation
GDPR	General Data Protection Regulation
PII	Personally Identifiable Information
